# Comparative analysis of the detection of antibiotic genotypic resistance with gastric mucosa, gastric fluid, and fecal samples in patients with *Helicobacter pylori* infection

**DOI:** 10.1128/jcm.01034-24

**Published:** 2024-12-16

**Authors:** Xinlu Ren, Baojun Suo, Cailing Li, Guangjie Ping, Lingling Ma, Yanyan Shi, Kai Zhou, Yuxin Wang, Xueli Tian, Liya Zhou, Zhiqiang Song

**Affiliations:** 1 Department of Gastroenterology, Peking University Third Hospital 66482 , Beijing, China; 2 Research Center of Clinical Epidemiology, Peking University Third Hospital 66482 , Beijing, China; Johns Hopkins University, Baltimore, Maryland, USA

**Keywords:** *Helicobacter pylori*, genotypic antibiotic resistance, gastric mucosa, gastric fluid, feces

## Abstract

Genotypic methods for detecting antibiotic resistance in *Helicobacter pylori* infection offer a rapid, convenient, and accurate approach for tailored therapy. However, existing studies predominantly examine single sample types and lack comparative analyses across different samples. This study comprehensively detects and compares genotypic resistance to clarithromycin and levofloxacin in gastric mucosa, gastric fluid, and fecal samples from the same patients. The study enrolled 183 participants, comprising 124 *H*. *pylori*-positive and 59 *H*. *pylori*-negative patients. All participants provided fecal samples and underwent gastroscopy for the collection of gastric mucosa and gastric fluid. Real-time PCR was employed to detect genotypic resistance to clarithromycin and levofloxacin in conjunction with bacterial culture and antibiotic susceptibility testing. Genotypic resistance detection rates for clarithromycin were 100% in gastric mucosa, 99.2% in gastric fluid, and 79.8% in fecal samples. For levofloxacin, detection rates were 97.6%, 96.8%, and 72.6%, respectively. The results showed that PCR detection for clarithromycin exhibited high sensitivity (0.94–0.95) and specificity (0.88–0.89) across all sample types. However, PCR detection for levofloxacin demonstrated slightly lower sensitivity (0.79–0.89) and specificity (0.79–0.83). The comparison of genotypic resistance results by PCR among the three sample types showed that gastric mucosa and gastric juice exhibited higher consistency, while the consistency between feces and both gastric mucosa and gastric juice was lower. This study confirmed good consistency between genotypic and phenotypic resistance in clarithromycin and levofloxacin. While both gastric mucosa and gastric fluid samples demonstrated high detection performance, the efficiency of detecting fecal samples was constrained by challenges in DNA extraction.

**IMPORTANCE:**

This study, with a large sample size, comprehensively tested both *Helicobacter* pylori-negative and -positive patients, including rapid urease test, histopathological evaluation and staining, bacterial culture, susceptibility testing, and resistance gene mutation analysis. By simultaneously examining gastric mucosa, gastric juice, and fecal samples from the same individuals, we minimized confounding factors arising from different sample sources, ensuring the reliability of our results. This approach effectively delineated the differences and characteristics in detection performance among different sample types, offering crucial reference data for selecting appropriate detection samples and identifying areas for improvement. The findings revealed robust concordance between genotypic and phenotypic resistance, with both gastric mucosa and gastric juice samples demonstrating excellent detection performance. However, the efficiency of detecting resistance in fecal samples was hampered by challenges in DNA extraction.

## INTRODUCTION

*Helicobacter pylori* infection and its associated diseases, such as peptic ulcers, gastric cancer, chronic gastritis, and dyspepsia, represent significant global health concerns. Effectively eradicating *H. pylori* is crucial for preventing and treating these prevalent conditions (1). Consensus and guidelines from both domestic and international experts uniformly recommend proactive eradication of all *H. pylori* infections unless contraindicated (2, 3). However, a major clinical challenge currently faced is the increasing difficulty in achieving eradication, largely due to empirical treatment protocols that do not account for bacterial resistance. This oversight has led to a rapid rise in resistance to antibiotics like clarithromycin and levofloxacin, substantially reducing treatment efficacy (4). Future successful eradication strategies will necessitate personalized drug selection and treatment to mitigate antibiotic resistance and avoid overuse (5).

Traditional personalized treatments rely on bacterial culture and susceptibility testing, which are technically demanding, time-consuming, and often yield low success rates, limiting their widespread clinical applicability (6). Advances in molecular biology and gene detection technologies have introduced rapid, straightforward, and accurate methods for detecting antibiotic resistance in *H. pylori* treatment (7–9). These methods replace phenotypic resistance testing (based on bacterial culture and susceptibility) with genotypic resistance testing (detection of mutations associated with resistance). Current research demonstrates strong agreement between genotypic and phenotypic methods for clarithromycin in the 23S rRNA gene and levofloxacin in the gyrA gene, paving the way for broader clinical adoption of personalized *H. pylori* treatment (10).

Regarding sample sources for genotypic resistance testing, gastric mucosa, gastric fluid, and fecal samples each have distinct advantages and limitations. Gastric mucosa samples provide high accuracy as *H. pylori* colonizes directly on the mucosal surface, although obtaining them requires invasive endoscopic procedures and mucosal biopsies, which are complex and costly. Due to the potential uneven distribution of *H. pylori* in the stomach, multiple biopsies may be necessary to enhance accuracy (11). Gastric fluid samples offer a comprehensive representation of the gastric environment, as *H. pylori* from different mucosal sites can shed into the gastric fluid. While this method avoids the need for biopsies, it is susceptible to external contaminants like dietary *H. pylori* or oral plaque and still often requires endoscopic assistance for collection (12, 13). Fecal samples, in contrast, are non-invasive, cost-effective, and easy to obtain, making them clinically practical. However, their utility is hampered by the presence of diverse microbial populations in the gut, which can interfere with *H. pylori* detection. In addition, DNA extraction from fecal samples is challenging due to digestive processes and PCR amplification inhibitors, resulting in lower efficiency (14–17).

Although studies have addressed all three types of samples, the focus has predominantly been on gastric mucosa, with fewer investigations on fecal samples and minimal attention to gastric fluid. Among these, most genotypic assays rely on PCR-based methods employing various techniques to analyze the resulting amplicons. In contrast, among the culture-based methods, the agar dilution and Epsilometer test (Etest) are the most widely utilized due to their accuracy and reliability. This research indicates consistent and satisfactory detection performance; however, the vast majority of studies have only examined a single sample type (14–26). To date, no studies have concurrently compared all three sample types within the same patient cohort. Such comparative studies would clarify differences and detection performance characteristics in varied sample origins, thereby reducing confounding factors associated with sample variability. This information is crucial for optimizing sample selection protocols and identifying areas for enhancement.

Based on these considerations, this study conducted diagnostic experiments by concurrently assessing gastric mucosa, gastric fluid, and fecal samples from the same patients for genotypic resistance to clarithromycin and levofloxacin. The findings will be contrasted with phenotypic resistance (bacterial culture and susceptibility testing as the gold standard) to demonstrate consistency and assess the disparities and efficacy among different sample types.

## MATERIALS AND METHODS

### Study population and design

Patients scheduled for gastroscopy at our center for various indications (aged 18–75 years, both male and female) were recruited between October 2021 and December 2023. They included both *H. pylori*-positive and -negative individuals who agreed to participate actively in the study. Exclusion criteria encompassed (i) prior *H. pylori* eradication, (ii) gastrointestinal malignancies, (iii) history of esophageal or gastric surgery, (iv) severe underlying illnesses, (v) recent use of proton pump inhibitors or potassium-competitive acid blockers within 2 weeks, and bismuth or antibiotics within 4 weeks prior to enrollment, (vi) pregnancy or lactation, and (vii) refusal or inability to cooperate with the study.

### Study procedure

Participants underwent screening based on inclusion and exclusion criteria, with relevant data documented. Standardized gastroscopy was performed on all subjects, involving collection of gastric fluid samples and gastric mucosal biopsies for rapid urease testing (RUT), histopathological assessment, Warthin-Starry staining, genotypic resistance testing, and bacterial culture with susceptibility testing. Fecal samples were collected within 3 days before or after gastroscopy. Subsequent tests and analyses were conducted, followed by data evaluation.

### *H. pylori* detection

*H. pylori* infection was confirmed by positive results in both RUT and gastric mucosal histological Warthin-Starry staining. Negative *H. pylori* status was determined by negative outcomes in both tests. Cases with discordant RUT and Warthin-Starry results were excluded from the study.

### Gastric fluid sample collection and processing

Between 3 and 5 mL of fasting gastric fluid specimens (with a minimum of 1mL) were collected during gastrointestinal endoscopy. The gastric fluid was directly suctioned into a sample container (KB8013-D, Suzhou Kebang Polymer Medical Apparatus Co., Ltd) via the endoscope’s suction base. After allowing the gastric fluid samples to settle and removing impurities, 0.5mL of gastric fluid was mixed with 1.0mL of Tris-HCl buffer (pH 8.0) and thoroughly vortexed. The mixture was then centrifuged at 12,000rpm for 3 minutes. Post-centrifugation, 200µL of the supernatant was used for subsequent DNA extraction. DNA extraction from gastric fluid was performed using the QIAamp DNA Mini Kit (order number 51306, Qiagen, Hilden, Germany).

### Rapid urease test

During the endoscopic examination, gastric mucosal samples from the antrum and body were obtained using biopsy forceps and placed into a single testing cup for the rapid urease test (HPUT-H102, San Qiang Bio & Che, Fujian, China). The procedure involved adding an enzyme reaction solution to the test cup to dissolve the reagent membrane completely. Gastric mucosal samples were then immersed in the reaction solution using a sterile specimen stick and incubated at 10°C–30°C for at least 5 minutes. The color change was visually assessed against a white paper background under natural light. A yellow solution indicated a negative result, suggesting no *H. pylori* infection, whereas a light red or rose-red solution indicated a positive result, confirming *H. pylori* infection. The results were evaluated by an experienced observer blinded to clinical details.

### Gastric mucosa sample collection and processing

Following the rapid urease test, two gastric mucosal samples were placed into an EP tube and sent to the testing center for genotypic analysis. DNA extraction from gastric biopsy specimens was conducted using the QIAamp DNA Mini Kit (order number 51306, Qiagen, Hilden, Germany).

### Gastric mucosa histological evaluation and Warthin-Starry staining

Three gastric mucosa samples were obtained from the gastric antrum, angular notch, and corpus, with additional biopsies taken if lesions were present. The gastric mucosa specimens were evaluated using the updated Sydney System and Chinese guidelines for the diagnosis and treatment of chronic gastritis, classifying chronic gastritis into atrophic and non-atrophic gastritis (27–29). Biopsy specimens for pathology underwent examination by an experienced pathologist after Warthin-Starry staining.

### *H. pylori* isolates culture and antimicrobial susceptibility testing

Only gastric mucosa samples from the antrum and corpus were placed into culture bottles (Brain Heart Infusion broth loaded with 20% glycerol) for *H. pylori* culturing and antimicrobial sensitivity testing (30). The gastric mucosa was homogenized, and the tissue fluid was inoculated onto *H. pylori* selective culture media. The *H. pylori* selective culture medium is a self-made medium, including Columbia agar (OxoidTM) with equine blood 7%, *H. pylori* selective supplement, and antibiotics (Dent 2%, OxoidTM). The inoculated plates were then incubated at 37°C in a mixed gas incubator (5% O_2_, 10% CO_2_, and 85% N_2_) for 3–11 days. *H. pylori* growth was monitored, and needle-like, translucent colonies were identified microscopically. Positive cultures were confirmed based on standard criteria, while cultures showing no growth after 11 days were considered negative or failed. *In vitro* clarithromycin and levofloxacin resistance was assessed using the Etest (AB Biodisk). A 100µL aliquot of the bacterial suspension was evenly spread onto Mueller-Hinton agar plates supplemented with 7% defibrinated horse blood using a sterile cotton swab. The density used was 2.0 McFarland standard (containing 1×10^7^–1×10^8^ CFU/mL). After the inoculum had been absorbed into the agar, Etest strips were placed on the agar surface using sterile tweezers. Plates were then incubated at 37°C under microaerophilic conditions for 72 hours, after which minimum inhibitory concentration (MIC) values were recorded. Phenotypic resistance was determined with thresholds set at>0.25 mg/L for clarithromycin and>1mg/L for levofloxacin according to the European Committee on Antimicrobial Susceptibility Testing breakpoints (EUCAST, version 14.0, 2024) (31).

### Fecal sample collection and processing

For accurate stool sample collection, samples classified as type III to V on the Bristol Stool Chart were selected. The stool sample was neither too liquid nor too solid, while also excluding abnormal components such as blood, mucus, or pus, which could affect test results. Stool collection was conducted using a sterile stool collection spoon to prevent microbial contamination. During collection, the spoon was not allowed to touch other surfaces or objects to minimize contamination risk. The optimal time for collection is the individual’s first bowel movement in the morning, which minimizes potential impacts of diarrhea and ensures sample freshness and integrity. Approximately the size of a walnut (at least 1gram) of stool should be collected and placed in a designated specimen collection cup. In the laboratory, approximately 200 milligrams of stool is used for DNA extraction. Collected samples should be stored in a preservation tube with a preservative solution to maintain integrity. DNA extraction from stool was performed using the QIAamp Fast DNA Stool Mini Kit (order number 51604, Qiagen, Hilden, Germany) following the manufacturer’s recommendations.

### Real-time PCR for genotypic resistance assessment

Gastric mucosal, gastric fluid, and fecal specimens were collected to detect mutations in resistance genes for clarithromycin and levofloxacin using real-time PCR (*Helicobacter pylori* 23S rRNA and gyrA genotyping test kit, Suzhou Changbo Biotechnology Co., China). Real-time PCR targeted the *ureB* gene to detect *H. pylori* presence. Specific resistance gene mutations were identified: A2142G, A2142C, and A2143G on the 23S rRNA gene for clarithromycin resistance, and mutations at positions 261, 271, and 272 on the gyrA gene for levofloxacin resistance. The reaction system had a total volume of 20µL, consisting of 0.6µL of 10µM specific primers and 0.4µL of fluorescence-labeled probes. The amplification protocol included an initial heating step at 95°C for 10 minutes, followed by a 2-minute annealing step at 37°C. Subsequently, 45 cycles of PCR amplification were performed, with each cycle consisting of denaturation at 95°C for 15 seconds, and annealing and extension at 60°C for 35 seconds. This detection system provided information on *H. pylori* infection presence, clarithromycin resistance, and levofloxacin resistance. Detection of both sensitive and resistant strains concurrently, indicating mixed infection, was interpreted as resistant. The sequences of the primers and probes used in this study are based on the previous literature (13, 32).

### Statistical analysis

Statistical analysis was conducted using SPSS Statistics 26, with significance set at two-sided *P* < 0.05. Categorical variables were presented as percentages or frequencies, while continuous variables were expressed as mean ± standard deviation. MedCalc software (version 22.016) was employed to calculate the area under the receiver operator characteristic curve (AUC), sensitivity, specificity, positive predictive value (PPV), negative predictive value (NPV), accuracy rate, and their 95% confidence intervals (33). The kappa coefficient assessed agreement between genotypic resistance and phenotypic resistance, with values of 0.6–0.8 indicating moderate agreement, 0.8–0.9 strong agreement, and>0.9 almost perfect agreement (34).

## RESULTS

### Patient enrollment and baseline data

**Fig 1:**
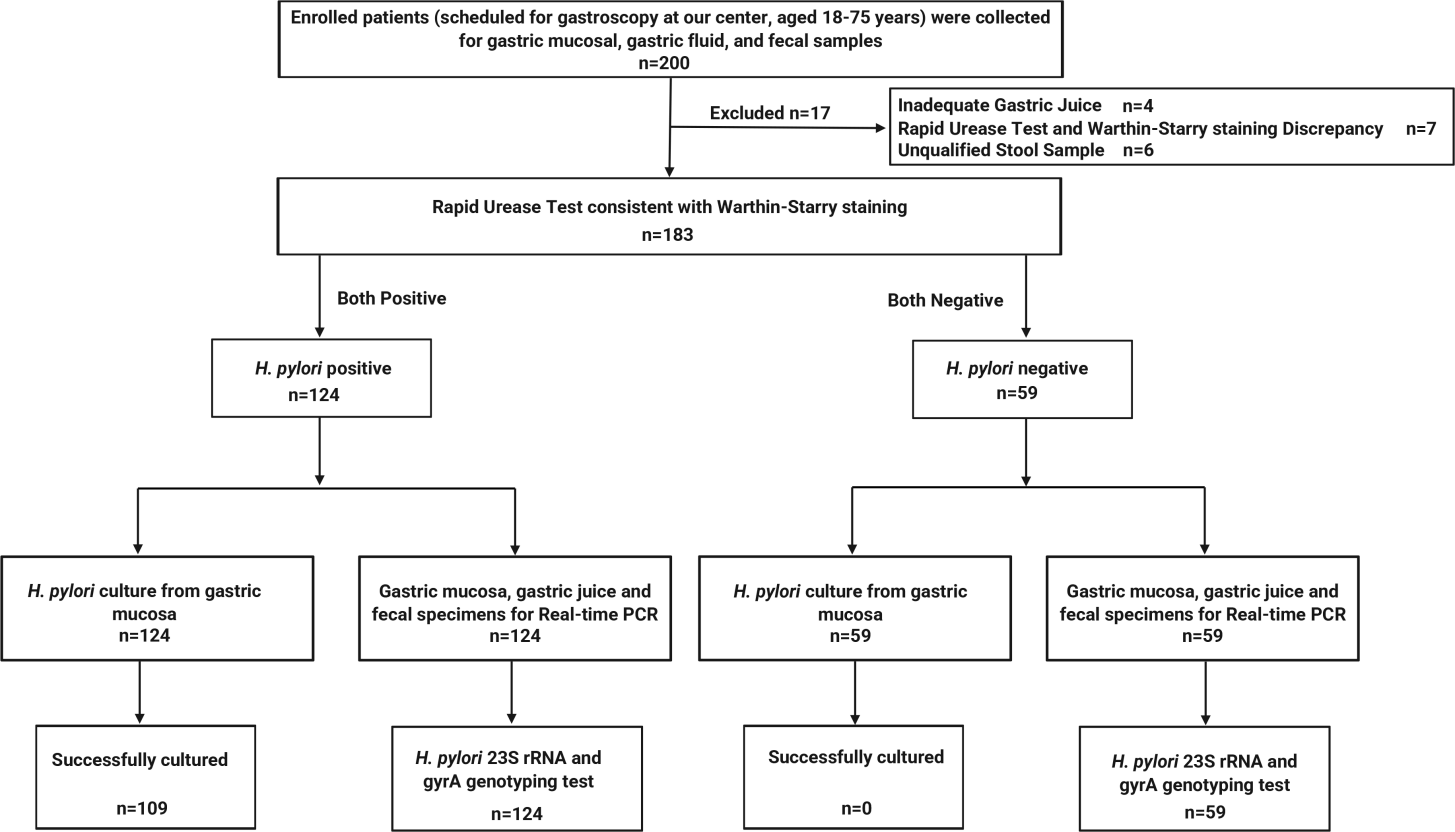
Flowchart of the study.

The patient flowchart is depicted in Fig. 1. A total of 200 patients were enrolled, all of whom underwent gastroscopy and provided gastric mucosal, gastric fluid, and fecal samples. Seventeen patients were excluded due to inadequate fecal (*n* = 6) or gastric fluid samples (*n* = 4), or discrepancies (*n* = 7) between RUT and Warthin-Starry staining results. Ultimately, 183 patients were included in the final comparative analysis, comprising 124 *H*. *pylori*-positive and 59 *H*. *pylori*-negative patients. All patients underwent bacterial culture (from gastric mucosa specimens) and real-time PCR testing from all sample types. Baseline characteristics of *H. pylori*-positive and *H. pylori*-negative patients are detailed in Table 1.

**TABLE 1:** Baseline characteristics

Patient characteristics	*H. pylori* infection status	
	No (*n* = 59)	Yes (*n* = 124)
Gender (male/female)	29/30	56/68
Age (years), mean ± SD	48.8 ± 13.7	44.2 ± 12.9
Diagnosis (no gastritis/chronic non-atrophic gastritis/chronic atrophic gastritis)	6/27/26	0/39/85
Success rate of *H. pylori* culture (from gastric mucosa specimens)	0	87.9%

### Diagnosis of *H. pylori* infection by real-time PCR

Among the 124 patients with *H. pylori* infection, real-time PCR confirmed *H. pylori* positivity in all 124 gastric mucosal samples (100%), all 124 gastric fluid samples (100%), and 102 fecal samples (82.3%). Among the 59 patients without *H. pylori* infection, 10 gastric mucosal samples (16.9%), 14 gastric fluid samples (23.7%), and 8 fecal samples (13.6%) tested positive.

### Acquisition of resistance gene information by real-time PCR

Among the 124 patients infected with *H. pylori*, resistance gene information was obtained through real-time PCR as follows: (i) clarithromycin resistance: yielded in all 124 gastric mucosal samples (100%), 123 gastric fluid samples (99.2%), and 99 fecal samples (79.8%). (ii) Levofloxacin resistance: yielded in 121 gastric mucosal samples (97.6%), 120 gastric fluid samples (96.8%), and 90 fecal samples (72.6%).

### Analysis of resistance rates

Resistance rate analysis was conducted on patients with either phenotypic or genotypic resistance information (Table 2): (i) clarithromycin resistance: the phenotypic resistance rate was 39.4%. Genotypic resistance rates detected in gastric mucosal, gastric fluid, and fecal samples were 46.0%, 46.3%, and 43.4%, respectively. (ii) Levofloxacin resistance: the phenotypic resistance rate was 33.0%. Genotypic resistance rates detected in gastric mucosal, gastric fluid, and fecal samples were 45.5%, 44.2%, and 41.1%, respectively. Genotypic resistance rates across the three sample types were comparable and consistently higher than phenotypic resistance rates. Furthermore, genotypic resistance testing was feasible even in the *H. pylori* culture-negative group.

**TABLE 2:** Phenotypic and genotypic resistance rates

Resistance rate	Phenotypic resistance	Genotypic resistance								
		Gastric mucosa PCR			Gastric fluid PCR			Fecal PCR		
		Overall	Culture-positive group	Culture-negative group	Overall	Culture-positive group	Culture-negative group	Overall	Culture-positive group	Culture-negative group
Clarithromycin	39.4% (43/109)	46.0% (57/124)	45.0% (49/109)	53.3% (8/15)	46.3% (57/123)	45.4% (49/108)	53.3% (8/15)	43.4%(43/99)	41.6% (37/89)	60.0% (6/10)
Levofloxacin	33.0% (36/109)	45.5% (55/121)	44.3% (47/106)	53.3% (8/15)	44.2% (53/120)	42.9% (45/105)	53.3% (8/15)	41.1%(37/90)	38.8% (31/80)	60.0% (6/10)

### Concordance analysis of phenotypic and genotypic resistance

Concordance analysis was performed on patients with available phenotypic and genotypic resistance data (see Table 3). The findings indicated the following: (i) clarithromycin: there was a high level of concordance between phenotypic and genotypic resistance, with an observed agreement of approximately 90% and a Kappa value of around 0.8, indicating substantial agreement. (ii) Levofloxacin: moderate concordance was observed between phenotypic and genotypic resistance, with an agreement rate of approximately 80% and a Kappa value of around 0.6, indicating a moderate level of agreement. Table 4 further details the diagnostic performance of the three sample types for detecting resistance.

**TABLE 3:** Concordance between phenotypic and genotypic resistance

Concordance analysis		Genotypic resistance					
		Gastric mucosa PCR		Gastric fluid PCR		Fecal PCR	
		Resistant	Sensitive	Resistant	Sensitive	Resistant	Sensitive
Clarithromycin phenotypic resistance	Resistant	41	2	41	2	31	2
	Sensitive	8	58	8	57	6	50
Observed agreement (%)		90.8		90.7		91.0	
Kappa value		0.81		0.81		0.81	
Levofloxacin phenotypic resistance	Resistant	32	4	31	5	22	6
	Sensitive	15	55	14	55	9	43
Observed agreement (%)		82.1		81.9		81.3	
Kappa value		0.63		0.62		0.60	

**TABLE 4:** Diagnostic ability of PCR for resistance detection in three sample types

Diagnostic ability	Sensitivity	Specificity	PPV	NPV	AUC
Clarithromycin					
Gastric mucosa	0.95 (0.84–0.99)	0.88 (0.78–0.95)	0.84 (0.73–0.91)	0.97 (0.88–0.99)	0.92 (0.85–0.96)
Gastric fluid	0.95 (0.84–0.99)	0.88 (0.77–0.95)	0.84 (0.73–0.91)	0.97 (0.88–0.99)	0.92 (0.85–0.96)
Feces	0.94 (0.80–0.99)	0.89 (0.78–0.96)	0.84 (0.71–0.92)	0.96 (0.87–0.99)	0.92 (0.84–0.96)
Levofloxacin					
Gastric mucosa	0.89 (0.74–0.97)	0.79 (0.67–0.87)	0.68 (0.57–0.77)	0.93 (0.84–0.97)	0.84 (0.75–0.90)
Gastric fluid	0.86 (0.71–0.95)	0.80 (0.68–0.88)	0.69 (0.58–0.78)	0.92 (0.83–0.96)	0.83 (0.74–0.90)
Feces	0.79 (0.59–0.92)	0.83 (0.70–0.92)	0.71 (0.57–0.82)	0.88 (0.78–0.94)	0.81 (0.70–0.89)

### Concordance analysis of genotypic resistance detection by PCR in three sample types

Concordance analysis was conducted on the genotypic resistance detection results obtained from gastric mucosa, gastric fluid, and fecal samples (see Tables 5 and 6). The findings revealed that for both clarithromycin and levofloxacin, there was a high concordance between the detection results from gastric mucosa and gastric fluid samples. However, the concordance between these samples and fecal samples was comparatively lower.

**TABLE 5:** Distribution of PCR detection results across three sample types^*a*^

	Gastric mucosa PCR	Gastric fluid PCR	Fecal PCR	Number of cases
Clarithromycin resistance	S	S	S	53
	R	R	R	40
	R	R	NA	14
	S	S	NA	10
	S	S	R	2
	R	R	S	2
	S	R	S	1
	S	NA	NA	1
	R	S	R	1
Levofloxacin resistance	S	S	S	48
	R	R	R	36
	S	S	NA	17
	R	R	NA	13
	R	R	S	4
	NA	NA	NA	3
	R	NA	NA	1
	R	S	S	1
	S	S	R	1

^*a*^ S, sensitive; R, resistant; NA, not available.

**TABLE 6:** Concordance analysis of PCR detection results across three sample types

Concordance analysis	Gastric mucosa sample vs gastric fluid sample		Gastric mucosa sample vs fecal sample		Gastric fluid sample vs fecal sample	
	Observed agreement rate (%)	Kappa value	Observed agreement rate (%)	Kappa value	Observed agreement rate (%)	Kappa value
Clarithromycin genotype resistance	97.6	0.95	76.7	0.61	75.8	0.60
Levofloxacin genotype resistance	98.4	0.97	70.1	0.53	71.8	0.55

## DISCUSSION

The rapid evolution of gene detection technologies has provided rapid, straightforward, and accurate methods for genotypic resistance testing, supplanting traditional bacterial culture, and susceptibility testing. This advancement has greatly facilitated the personalized selection of eradication treatment of *H. pylori* infections in clinical settings. In this study, we employed the real-time PCR method, commonly used for infectious disease detection and resistance gene amplification. This method targets mutation sites in clarithromycin and levofloxacin resistance genes, offering high sensitivity, accuracy, cost-effectiveness, and operational simplicity, thereby making it suitable for widespread clinical application (13–15, 21, 25, 35).

Numerous diagnostic studies have compared genotypic resistance with phenotypic resistance using various single detection samples, with gastric mucosal samples being the most extensively researched. Compared to phenotypic resistance, genotypic resistance detection in gastric mucosal samples demonstrates very high sensitivity and specificity. A meta-analysis by Wang et al. (36) indicated that the sensitivity and specificity in detecting clarithromycin and levofloxacin genotypic resistance in gastric mucosal samples exceed 95%. Research on gastric fluid samples is relatively sparse, yet existing studies suggest satisfactory sensitivity and specificity, particularly for clarithromycin (over 90%), albeit lower than that of gastric mucosal samples (12, 13, 23). Fecal samples have garnered attention due to their ease of collection. Our published meta-analysis demonstrated that in DNA-extractable fecal samples, sensitivity and specificity for clarithromycin genotypic resistance detection were 93% and 98%, respectively. However, DNA extraction from fecal samples often presents challenges (37, 38). First, *H. pylori* nucleic acid content in feces is exceedingly low and prone to degradation. Second, abundant intestinal microbial nucleic acids can interfere with the amplification process (39). Moreover, impurities in fecal samples can significantly impair the efficiency of real-time PCR amplification, necessitating their removal during the extraction process to minimize interference. There have also been reports on using saliva and dental plaque samples for detection. However, due to low DNA extraction rates and poor sensitivity and specificity, such studies have become less prevalent (22, 40).

In the current research, studies utilizing gastric fluid and fecal samples for detection remain relatively limited, particularly those simultaneously examining different sample types from the same individual. Such studies can mitigate confounding factors, enabling a more robust comparison and comprehension of the strengths and limitations of various detection samples. This approach can guide the rational selection of detection samples and highlight areas requiring enhancement. Based on this rationale, our study was conducted.

In this study, real-time PCR was used to diagnose *H. pylori* infection, with both gastric mucosal and gastric fluid samples consistently detecting the infection in all positive cases, while a minority of fecal samples yielded negative results. This discrepancy mainly stemmed from challenges in extracting DNA from fecal samples. In the infection-negative group, a few positive results were noted across all sample types, possibly due to transient *H. pylori* strains, laboratory variables, or the high sensitivity of PCR detection.

Regarding the acquisition of genotypic resistance information via PCR, both clarithromycin and levofloxacin resistance data were reliably obtained from gastric mucosal and gastric fluid samples. However, a small fraction of fecal samples could not undergo genotypic resistance testing due to DNA extraction difficulties. Initially, a column-based method was used for DNA extraction from fecal samples, but the yield and specificity were suboptimal. Recently, we have explored a magnetic bead method, which employs magnetic beads to bind DNA molecules, facilitating separation using an external magnetic field. Preliminary findings indicate an extraction rate of approximately 90%, promising improved detection performance for fecal samples in future studies (37, 41, 42).

In terms of resistance detection, this study underscores two advantages of the PCR method. First, its high sensitivity enables genotypic resistance detection even in *H. pylori*-infected individuals who test negative by culture. Second, it can identify mixed-strain resistance, where individuals harbor both sensitive and resistant mutant genes. In contrast, bacterial culture coupled with susceptibility testing only reflects the resistance status of the dominant strain, potentially underestimating actual resistance levels. As long as DNA extraction is feasible, genotypic resistance detection remains consistent across all three sample types.

Regarding concordance analysis between phenotypic and genotypic resistance, the findings demonstrate good agreement across gastric mucosa, gastric fluid, and fecal samples, particularly for clarithromycin, albeit slightly lower for levofloxacin. This suggests that, given successful DNA extraction, real-time PCR for genotypic resistance detection from these sample types effectively substitutes bacterial culture and susceptibility testing in reflecting clarithromycin and levofloxacin resistance. A pairwise comparison of genotypic resistance detection among gastric mucosa, gastric fluid, and fecal samples revealed high concordance between gastric mucosa and gastric fluid, with lower concordance involving fecal samples, primarily due to DNA extraction challenges.

Through the comparative study mentioned above, we have gained a clearer understanding of the advantages and disadvantages of using different samples. The results affirm that gastric mucosa samples offer excellent accuracy and reliability for detection. To mitigate the variability in *H. pylori* distribution within the stomach, we collected mucosal tissues from both the gastric body and antrum, combining them for PCR testing, which demonstrated very good detection performance. However, the requirement for endoscopy and mucosal biopsy, both invasive and traumatic procedures, limits the widespread clinical application of gastric mucosa samples.

Gastric fluid samples have been less studied, but our findings suggest they perform comparably to gastric mucosa samples in genotypic resistance detection, showing high concordance in detection rates and consistency. Since gastric fluid samples better reflect overall gastric conditions and do not necessitate mucosal biopsy, they could potentially replace gastric mucosa samples for genotypic resistance testing. Simplifying gastric fluid collection methods, such as through the string test or simple gastric fluid collectors, would greatly enhance their clinical utility (43, 44).

Fecal samples offer the greatest clinical utility due to their simplicity, low cost, and non-invasive nature. The primary challenge lies in improving the extraction of sufficient *H. pylori* DNA for resistance gene detection. Once adequate DNA extraction is achieved, fecal samples can provide detection performance comparable to that of gastric mucosa and gastric fluid samples. Improving amplification efficiency and reducing inhibitory factors in fecal samples are critical steps toward enhancing detection performance.

This study, with a large sample size, comprehensively tested both *H. pylori*-negative and -positive patients, including RUT, histopathological evaluation and staining, bacterial culture, susceptibility testing, and resistance gene mutation analysis. By simultaneously examining gastric mucosa, gastric fluid, and fecal samples from the same individuals, we minimized confounding factors arising from different sample sources, ensuring the reliability of our results. This approach effectively delineated the differences and characteristics in detection performance among different sample types, offering crucial reference data for selecting appropriate detection samples and identifying areas for improvement. Such a comprehensive methodology has not been previously explored in the literature.

This study has several limitations. First, being a single-center study, the generalizability of the results may be limited, and future research should aim to validate these findings in diverse regions and populations. Second, we exclusively assessed genotypic resistance to clarithromycin and levofloxacin, without evaluating other antibiotics. This limitation stems from the current inadequate agreement between genotypic and phenotypic resistance for other antibiotics used in *H. pylori* eradication. Contributing factors include the dispersed distribution and low representation of resistance gene mutation sites, alongside non-genetic mechanisms of resistance (45, 46). Our kit targets key point mutations instead of all possible ones, as these core point mutations cover most cases. Studies both domestically and internationally also focus on these common point mutations, and detecting resistance to clarithromycin and levofloxacin captures the majority of cases (47–50). Future efforts should concentrate on developing genotypic resistance detection methods for a wider array of antibiotics to offer broader options for individualized clinical treatment. Third, seven patients with discordant RUT and Warthin starry were excluded from the study. In most studies on *H. pylori* infection, the infection status is determined by consistent results from at least two common tests (such as RUT, urea breath test, histopathological staining, or fecal antigen test). If both tests are positive, the patient is considered infected; if they are negative, infection is ruled out. Patients with inconsistent results are excluded to avoid false positives or negatives. When the results of the two methods are inconsistent, the infection status can be further determined by other methods or repeating the test at intervals after ruling out possible interfering factors in the future.

Finally, this study included only treatment-naive patients with *H. pylori* infection. This selection criterion is due to the relatively low resistance rates to clarithromycin and levofloxacin in treatment-naive patients, contrasting with significantly higher rates in retreatment patients due to secondary and cross-resistance phenomena (51). Consequently, the clinical utility of genotypic resistance testing for these antibiotics primarily applies to treatment-naive patients.

In conclusion, this study employed real-time PCR technology to assess genotypic resistance to clarithromycin and levofloxacin, conducting comprehensive analyses and comparisons of gastric mucosa, gastric fluid, and fecal samples from the same individuals. The findings revealed robust concordance between genotypic and phenotypic resistance, with both gastric mucosa and gastric fluid samples demonstrating excellent detection performance. However, the efficiency of detecting resistance in fecal samples was hampered by challenges in DNA extraction. Improving DNA extraction techniques represents a crucial avenue for future research.
